# Efficacy and safety of artemisinin-naphthoquine *versus* dihydroartemisinin-piperaquine in adult patients with uncomplicated malaria: a multi-centre study in Indonesia

**DOI:** 10.1186/1475-2875-11-153

**Published:** 2012-05-03

**Authors:** Emiliana Tjitra, Armedy R Hasugian, Hadjar Siswantoro, Budi Prasetyorini, Riyanti Ekowatiningsih, Endah A Yusnita, Telly Purnamasari, Srilaning Driyah, Ervi Salwati, Nurhayati, Eni Yuwarni, Lidwina Januar, Joseph Labora, Bambang Wijayanto, Fajar Amansyah, Tersila AD Dedang, Asep Purnama, Trihono

**Affiliations:** 1 National Institute of Health Research and Development, Ministry of Health, Jakarta, Indonesia; 2 Centre for Biomedical Research and Development, Jayapura, Papua, Indonesia; 3 Marthen Indeys Army Hospital, Jayapura, Papua, Indonesia; 4 Soedibjo Sardadi Navy Hospital, Jayapura, Papua, Indonesia; 5 Bhayangkara Police Hospital, Jayapura, Papua, Indonesia; 6 Kewapante Catholic Hospital, Maumere, Sikka, East Nusa Tenggara, Indonesia; 7 TC Hillers District Public Hospital, Maumere, Sikka, East Nusa Tenggara, Indonesia

**Keywords:** Malaria, Artemisinin-naphthoquine, Dihydroartemisinin-piperaquine, Efficacy, Safety

## Abstract

**Background:**

A practical and simple regimen for all malaria species is needed towards malaria elimination in Indonesia. It is worth to compare the efficacy and safety of a single dose of artemisinin-naphthoquine (AN) with a three-day regimen of dihydroartemisinin-piperaquine (DHP), the existing programme drug, in adults with uncomplicated symptomatic malaria.

**Methods:**

This is a phase III, randomized, open label using sealed envelopes, multi-centre, comparative study between a single dose of AN and a three-day dose of DHP in Jayapura and Maumere. The modified WHO inclusion and exclusion criteria for efficacy study were used in this trial. A total of 401 eligible adult malaria subjects were hospitalized for three days and randomly treated with AN four tablets single dose on day 0 or DHP three to four tablets single daily dose for three days, and followed for 42 days for physical examination, thick and thin smears microscopy, and other necessary tests. The efficacy of drug was assessed by polymerase chain reaction (PCR) uncorrected and corrected.

**Results:**

There were 153 *Plasmodium falciparum*, 158 *Plasmodium vivax* and 90 *P. falciparum/P. vivax* malaria*.* Mean of fever clearance times were similar, 13.0 ± 10.3 hours in AN and 11.3 ± 7.3 hours in DHP groups. The mean of parasite clearance times were longer in AN compared with DHP (28.0 ± 11.7 hours *vs* 25.5 ± 12.2 hours, p = 0.04). There were only 12 PCR-corrected *P. falciparum* late treatment failures: seven in AN and five in DHP groups. The PCR uncorrected and corrected on day −42 of adequate clinical and parasitological responses for treatment of any malaria were 93.7% (95% Cl: 90.3–97.2) and 96.3% (95% Cl: 93.6–99.0) in AN, 96.3% (95% Cl: 93.5–99.0) and 97.3% (95% Cl: 95.0–99.6) in DHP groups. Few and mild adverse events were reported. All the abnormal haematology and blood chemistry values had no clinical abnormality.

**Conclusion:**

AN and DHP are confirmed very effective, safe and tolerate for treatment of any malaria. Both drugs are promising for multiple first-line therapy policies in Indonesia.

## Background

Malaria remains a major public health problem. The World Health Organization (WHO) estimated the number of reported cases from Indonesia were 2.5 million in 2006 [1]. Most cases were recorded from Papua and East Nusa Tenggara provinces, where about 300,000 and 70,000 clinical malaria cases were reported annually from its provinces. To accelerate malaria control, one of the four key recommended interventions is to give appropriate anti-malarial drugs with artemisinin-based combination therapy (ACT) for patients with confirmed malaria [1].

Artemisinin was discovered by Chinese scientists in the 1970s. Artemisinin derivatives are the most rapidly acting and efficacious anti-malarial drugs. These drugs show rapid absorption and activity against many stages of *Plasmodium*, including trophozoites and early sexual forms (gametocytes). Their short elimination half-life (<3.7 hours) protects them from resistance and their potent and broad specificity of action reduces gametocyte carriage and infectivity, and reduces the transmission of *Plasmodium falciparum*. Tolerability of these drugs is also very good [2,3]. This makes artemisinin derivatives the ideal partner drugs. WHO recommends the use of artemisinins only in combination with another anti-malarial drug to prevent the occurrence of drug resistance and to address the issue of its relatively short half-life. ACT is the best therapeutic option for treating drug-resistant malaria and retarding the development of resistance presently [4].

Since 2004, the Indonesian Malaria Control Programme has chosen a non-fixed dose artesunate-amodiaquine (AA) as the programme drug for treatment of uncomplicated *P. falciparum,* which had widely reported resistant to chloroquine, sulphadoxine-pyrimethamine or quinine [5]. AA is also effective for *Plasmodium vivax*, safe for all age groups and relatively cheap. Though combination of AA is palatable and given by single daily dose for three days, the compliance is poor because of the number of pills to be swallowed. This ACT resulted a problem in its wide-scale implementation. Moreover, there was reported *in vitro* amodiaquine cross-resistance with chloroquine [5]. Consequently, the efficacy of AA was varied (78–96%) [6]. To overcome these problems, other ACT is needed for treatment of any malaria in Indonesia.

Fixed-dose combination of artemether-lumefantrine (AL) has been registered recently in Indonesia. This ACT was safe and very effective with a cure rate of 95% for *P. falciparum* malaria, but its efficacy was modest (43%) for *P. v*ivax [7]. AL is not a practical regimen because it should be administered twice daily for three days and given with fatty food. Moreover, the cost is expensive, >10 US$ per treatment course. A better ACT that would be simple to use, effective and affordable for all types of malaria is needed to eliminate malaria in Indonesia.

Dihydroartemisinin-piperaquine (DHP) is a fixed-dose ACT and given single daily dose for three days. Clinical trials for treatment of uncomplicated malaria proved more superior compared with the existing fixed-dose AL, and ACT programme AA in Papua. This ACT is very effective with cure rates of ≥95% for all malaria and safe [6,7]. DHP has been used widely as the first-line ACT for more than two years in Papua. In addition, the cost of DHP is similar to AA per treatment course. DHP trials in other areas are needed to collect unexpected adverse events.

Artemisinin-naphthoquine (AN) is a new fixed-dose ACT. Naphthoquine phosphate is an anti-malarial drug synthesized by Chinese Academy of Military Medical Science in late 1980s. Though naphthoquine has a similar structure to chloroquine, cross-resistance has not yet been reported. Existing pharmacological data indicate that naphthoquine is effective against erythrocytic phase of *Plasmodium*, even in chloroquine resistance cases. Naphthoquine has a longer half-life (276 hours), compared to chloroquine and mefloquine. It has been shown to be effective against *P. falciparum* at doses of 12 mg/kg used alone or at 400 mg in combination with artemisinin for adult patients. Naphthoquine appears to be an ideal partner drug for artemisinin [8].

AN is administered with a single dose of therapy and has few side effects. Of limited data, AN was reported safe and effective for both *P. falciparum* and *P. vivax *[9,10]. In a study in Chinese adult subjects, one dose of a fixed-dose artemisinin (1,000 mg)-naphthoquine (400 mg) for treatment of uncomplicated malaria showed a cure rate of 98% at day 28 in *P. falciparum*, and 90% at day 56 in *P. vivax *[11]. More studies should be done to confirm its efficacy, safety, and tolerability in an enlarge scale and another population.

Indonesia has been committed to eliminate malaria by year 2030. A clinical trial of AN was conducted to improve compliance and find a practical and simple ACT for all malaria species. Based on the previous Indonesian ACT trials, DHP is the best ACT for treatment of uncomplicated malaria in multi-drug resistant areas. Therefore, a clinical trial of AN was compared to DHP for its efficacy and safety in Jayapura and Maumere.

## Methods

### Time and study location

The trial was carried out in 2007–2008 at four hospitals, three Armed Forces hospitals in Jayapura (Marthen Indeys/Army, Soedibjo Sardadi/Navy and Bhayangkara/Police Hospitals), and one public hospital in Maumere (St Gabriel Hospital).

### Study design

The study was a phase III, randomized, open label, multi-centre, comparative of the efficacy, safety and tolerability of a single dose AN *vs* DHP in adults with uncomplicated symptomatic *P. falciparum*, *P. vivax* or mixed *P. falciparum*/*P. vivax* malaria. This trial was approved in writing by the Ethics Committee of National Institute of Health Research and Development, Ministry of Health (No. LB.03.02/2/449/2007), and the Bureau of Food and Drug Control (No.PO.01.01.3.1.1682), Republic of Indonesia.

### Sample size

Assuming the failure rates of AN (P_1_) and DHP (P_2_) were 0.1% and 5.0% based on un-published African AN studies prior a trial and the WHO recommendation for choosing ACT programme [12]. The study estimated risk ratio of failure rate was 2% with α (type I error) of 0.05 and power (1-β) of 80% [13]. A minimum sample size was 166 subjects per treatment group, and adjusted 20% for follow-up losses and withdrawals. A total 401 subjects were recruited in this trial.

The formula for calculating the sample size as the following [13]

(1) $$\begin{array}{c} \text{N}=7.85\;\left[\left(\text{R}+1\right)-{\text{P}}_{2}\left({\text{R}}^{2}+1\right)\right]\\ /{\text{P}}_{2}{\left(1-\text{R}\right)}^{2}=166+20\%=200\;\text{subjects} \end{array}$$

N = The sample size of each of the treatment groups; P1 = The failure rate in the Arco™ group (0.1%); P2 = The failure rate in the Duo-Cotecxin™ group (5.0%); R = The risk ratio of treatment failure (P1/P2 = 0.02).

### Procedures

The population of this study was adult males and females aged 15–69 years, body weight 35–75 kg and presenting with acute, symptomatic, uncomplicated *P. falciparum* and/or *P. vivax* malaria. They were recruited according to the modified WHO inclusion criteria (absence of severe malnutrition, axillary temperature of ≥37.5°C or history of fever in the last 24 hours, asexual *P. falciparum* density 1,000–200,000/μl*, P. vivax* and other malaria density ≥250/μl, and ability to swallow oral medication), and exclusion criteria [severe vomiting, history or evidence of clinically systematic significant disorders, other febrile conditions, hypersensitivity or adverse reactions to anti-malarials, history of use of any other anti-malarial agent within four weeks prior to start of the study and confirmed by urine test (Dill Glazko and Lignin tests), and pregnancy or lactating] for therapeutic efficacy study [14]. Subject informed consent was requested prior the study. Subjects who withdrew early were not replaced.

Eligible subjects were blindly, randomly assigned equally to one of the two treatment groups using sealed envelopes. AN (Arco™, Kunming Pharmaceutical Corporation with Chinese quality standards, one tablet contained 250 mg of artemisinin and 100 mg of naphthoquine) was administered four tablets single dose only. DHP (Duo-Cotecxin™, Holey-Cotec Pharmaceutical Co.LTd, China, one tablet contained 40 mg of dihydroartemisinin and 320 mg of piperaquine) was administered three (body weight of ≤60 kg) to four tablets (body weight of >60 kg) single daily dose for three days based on dosage of dihydroartemisinin 2–4 mg/kg bw or piperaquine 16–32 mg/kg bw [6,7]. Subjects were observed for one hour to ensure that the medications were not vomited. All subjects were hospitalized for three days or until fever and parasite had cleared for at least 24 hours, returned to study site for follow-up at all scheduled visits to day 42, and they had additional primaquine for radical treatment on day 42. Subjects with treatment failure were withdrawn from the study and given a rescue malaria treatment [12]. They had no study investigations performed thereafter.

### Clinical and laboratory assessments

All eligible subjects had medical history and detail demography completed at enrolment. A full physical examination, electrocardiography (ECG) and laboratory tests (malaria microscopy, haematology, blood chemistry, PCR genotyping and urinalysis) were performed at baseline (day 0, prior to dose). Limited physical examination was performed during hospitalization (days 1–2) and on follow-up days (3, 7 14, 21, 28, 35, and 42) and if clinically indicated as well as adverse events collected each time. A 12-lead resting ECG was obtained approximately 2 to 4 hours after study drug administration on day 0–2, and follow up days 7, 28 and 42. Thick and thin smears were examined at screening, days 0–2 hospitalization: eight hourly, days 3, 7, 14, 21, 28, 35, and 42; haematology and blood chemistry at days 0, 3, 7, and 28; and blood spot for PCR at days 0 and 42 or failure. Urinalysis was assessed on days 0 and 3. HCG was tested for women of potential pregnancy at screening and day 28. Microscopy results were blind cross-checked by certified microscopists, and treatment failures were corrected by PCR. PCR was performed for speciation of plasmodium and genotype of *P. falciparum*. There were 3 loci genotype of *P. falciparum* tested (MSP1, MSP2 and GLURP) [15]. The primary (P) and nested (N) primers are as following : MSP1 (P1: 5′CAC ATG AAA GTT ATC AAG AAC TTG TC3′, P2: 3′GTA CGT CTA ATT CAT TTG CAC G5′; N1: 5′GCA GTA TTG ACA GGT TAT GG3′, N2: 3′GAT TGA AAG GTA TTT GAC5′); MSP2 (P1: 5′GAA GTT AAT TAA AAC ATT GTC3′, P2: 3′GAG GGA TGT TGC TGC TCC ACA G5′, N1: 5′CTA GAA CCA TGC ATA TGT CC3′, N2: 3′GAG TAT AAG GAG AAG TAT G5′) and GLURP (P1: 5′ACA TGC AAG TGT TGA TCC3′, P2: 3′GAT GGT TTG GGA GTA ACG5′, N1: 5′TGA ATT CGA AGA TGT TCA CAC TGA AC3′, N2: 3′TGT AGG TAC CAC GGG TTC TTG TGG5′). To date, there are no recommended markers to distinguish recrudescence, relapse and new infection of *P. vivax* malaria from *P. vivax* treatment failures.

### Efficacy assessment

The efficacy of AN and DHP was assessed in intent-to-treat (ITT), modified ITT, and evaluable or per-protocol (PP) population. ITT population included all randomized subjects who had received any amount of study medication. Modified ITT population only included correctly randomized subjects in analysis, and excluded wrongly randomized and those lost to follow-up [16]. PP analysis consisted only of the efficacy evaluable (EE) subjects defined according to the 2003 WHO criteria [14] and constituted as PP population that did not include subjects who failed to comply with per-protocol.

The efficacy was a proportion of subjects with PCR-corrected adequate clinical and parasitological response (ACPR) at day 42. ACPR was defined as the absence of asexual parasitaemia on day 42 irrespective of the temperature and not meeting any of criteria of early treatment failure (ETF) or late clinical or parasitological failure (LCF or LPF), or as subjects with clearance of asexual parasitaemia within 42 days of initiation of study treatment. Subjects classified as failures by clinical and parasitological criteria were considered ACPR if the PCR analysis showed a new infection (all the alleles in parasites from the failure-treatment sample were different from those in the admission sample, for one or more loci tested) rather than a recrudescence . Recrudescence was defined as reappearance of asexual parasites of the same isolate as initial infection with or without clinical signs, after initial clearance of parasites from the peripheral blood with positive blood smear and PCR confirmation of the same isolate (presence of at least one matching alleles) [15]. The early and late failures were classified according to the 2003 WHO guidelines. The total treatment failure was defined as the sum of early and late treatment failures [14].

### Safety assessment

The safety population was defined as all randomized subjects who had received any amount of study medication. Safety was assessed through direct questioning, physical examinations, ECG abnormalities (prolongation QT- interval), and significant change from baseline clinical laboratory parameters [17]. Adverse events were followed up until the event had resolved.

### Data analysis

Data were double entered and validated including microscopic validation and PCR corrected treatment failure data using EpiData 3.02, and analysed using SPSS for Windows version 15. The Mann–Whitney U-test was used for non-parametric comparisons, and Student’s t-test for parametric comparisons. Proportions were examined using *χ*^2^ with Yates’ correction or by Fisher’s exact-test. The efficacy was assessed by survival analysis in which the cumulative risk of failure was calculated by the Kaplan Meier product limit formula.

## Results

### Baseline characteristics of study subjects

In Jayapura, over 3,000 clinical malaria cases had been screened, only 301 could be enrolled for this trial, and 151 cases were treated with AN and 150 treated with DHP. In Maumere, of a total 154 screened clinical malaria, only 100 cases could participate, 50 were treated with AN and the other 50 treated with DHP. The Armed Forces Hospitals contributed to 75% sample size, so were mostly male subjects. There were seven subjects had weight >75 kgs (four in AN and three in DHP), 56% with axillary temperature of ≥37.5°C. The characteristics of study subjects in both treatment groups were not different (Table 1).

**Table 1:** Characteristics of study subjects by the study drug on enrolment

**Characteristic**	**Artemisinin- naphthoquine(AN)**	**Dihydroartemisinin- piperaquine(DHP)**	**P**	**Overall **
**Number of subjects**	**201**	**200**		**401**
**Age:mean ± SD (range) years**	27.6 ± 10.8 (15–69)	26.2 ± 9.2 (15–67)	0.18	26.9 ± 10.0 (15–69)
**Body weight: mean ± SD (range) kgs**	58.7 ± 8.5 (36–81)	58.5 ± 8.5 (37–85)	0.86	58.6 ± 8.5 (36–85)
**Axillary temperature: mean ± SD (range)** °**C**	37.9 ± 1.0 (36–40.3)	37.9 ± 1.1 (35–40.2)	0.84	37.9 ± 1.1 (35–40.3)
**Blood Pressure Systole: mean ± SD (range) mmHg**	113 ± 13 (90–150)	114 ± 13 (80–170)	0.44	113 ± 13 (80–170)
**Diastole: mean ± SD (range) mmHg**	72 ± 9 (50–92)	73 ± 9 (40–120)	0.39	72 ± 9 (40–120)
**Heart rate: mean ± SD (range) per min**	86 ± 13 (52–126)	87 ± 13 (56–126)	0.59	86 ± 13 (52–126)
**Respiration rate: mean ± SD (range) per min**	20 ± 3 (16–36)	20 ± 3 (16–32)	0.51	20 ± 3.0 (16–36)
**Sex: male:female (%)**	181:20 (90:10)	170:30 (85:15)	0.17	351:50 (87.5:12.5)
**History of fever in the last 24 hours (%)**	195 (97.0)	199 (99.5)	0.12	394 (98.3)
**Study subject with T.axillary ≥37.5**°**C (%)**	114 (56.7)	109 (54.5)	0.73	223 (55.6)
**History of experience malaria in the last year (%)**	160 (79.6)	159 (79.5)	1.00	319 (79.6)
**Frequency of malaria in the last year: mean ± SD (range) times**	2.6 ± 2.3 (1–20)	2.4 ± 1.6 (1–12)	0.53	2.7 ± 3 (1–20)
**History of taken antimalarial drugs in the last 4 weeks (%)**	55 (27.4)	57 (28.5)	0.89	112 (27.9)

**Figure 1:**
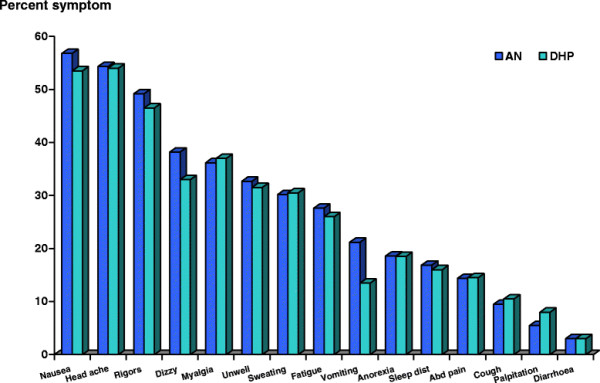
Proportions of malaria symptom and sign on enrolment in artemisinin-naphthoquine (AN) versus dihydroartemisinin-piperaquine (DHP) groups were no significant difference (p > 0.05).

The clinical symptoms of subjects in AN and DHP groups were also not different. Fever, nausea, headache and rigors were the common symptoms documented in this study. Other classical symptoms are shown on Figure 1.

The initial laboratory findings of haematology, blood chemistry, and parasitology showed no differences between AN and DHP groups (Table 2). Though some of the study subjects had abnormal values, only a few had significant clinical abnormalities. The distribution of malaria subjects with anaemia (Hb < 11 g/dL), thrombocytopaenia (platelet < 150,000/ul) or leucocytosis (>10,000/ul) were almost similar between the treatment groups (52.2% *vs* 47.8%, 71.6% *vs* 73%, 6% *vs* 5%). Overall, only pallor was documented as a significant clinical abnormality with abnormal values of haematology.

**Table 2:** Haematology, blood chemistry and parasitology findings of study subjects on enrolment by the study drug

**Parameter**	**Artemisinin- naphthoquine (AN)**	**Dihydroartemisinin- piperaquine (DHP)**	**P**	**Overall**
**Number of subjects**	**201**	**200**		**401**
**Haematocrit: mean ± SD (range)%**	36.1 ± 7.1 (15.5–68.9)	36.6 ± 6.6 (19.1–63.4)	0.46	36.4 ± 6.8 (15.5–68.9)
**Haemoglobin: mean ± SD (range) g/dL**	12.5 ± 2.3 (6.2–23.1)	12.6 ± 2.1 (6.6–21.1)	0.67	12.6 ± 2.2 (6.2–23.1)
**Red Blood Cell: mean ± SD (range) per uL**	4.5 ± 0.8 (1.5–7.7)	4.4 ± 0.8 (2.2–7.3)	0.70	4.4 ± 0.8 (1.5–7.7)
**Platelet: mean ± SD (range) 10**^**3**^**/mm**^**3**^	116.4 ± 68.7 (1.8–381.0)	116.5 ± 65.9 (1 .0–601.0)	0.98	116 ± 67.2 (1.0–601.0)
**White Blood Cell: mean ± SD (range) per uL**	6.4 ± 2.2 (2.0–14.0)	7.1 ± 6.0 (2.2–65.0)	0.11	6.8 ± 4.5 (2.0–65.0)
**Bilirubin: mean ± SD (range) mg/dL**	1.0 ± 0.6 (0.1–5.2)	1.0 ± 0.5 (0.1–3.8)	0.67	1.0 ± 0.5 (0.1–5.2)
**Albumin: mean ± SD (range) g/dL**	4.1 ± 1.0 (2.0–7.4)	4.2 ± 1.0 (2.2–6.2)	0.25	4.2 ± 1.0 (2.1–7.4)
**ALT (SGPT): mean ± SD (range) IU/L**	28.8 ± 13.0 (5–73)	28.3 ± 14.2 (6.9–98.0)	0.71	28.5 ± 13.9 (5.0–98.0)
**AST (SGOT): mean ± SD (range) IU/L**	29.4 ± 14.0 (2.0–87.0)	28.5 ± 11.8 (2.6–91.0)	0.49	28.9 ± 12.9 (2.0–91.0)
**Creatinine: mean ± SD (range) mg/dL**	1.0 ± 0.4 (0.2–2.5)	0.9 ± 0.3 (0.2–2.0)	0.15	0.9 ± 0.4 (0.2–2.5)
**Urea: mean ± SD (range) mg/dL**	27.5 ± 11.7 (2.7–80.0)	27.6 ± 11.7 (8.6–110.0)	0.94	27.6 ± 11.7 (2.7–110.0)
**Density of asexual parasites: geometric mean (range) per uL**	6310 (304–113550)	6972 (372–140084)	0.21	6634 (304–140084)
**Gametocyte carriages (%)**	134 (67.3)	140 (70.3)	0.54	274 (68.8)
**Density of gametocytes: geometric mean (range) per uL**	40 (1–2697)	35 (2–2208)	0.55	38 (1–2697)

Some study subjects had higher or lower values of blood chemistry, such as alanine aminotransferase (ALT) (23.4%), aspartate aminotransferase (AST) (25.9%), bilirubin (31.4%), albumin (44.6%), urea (5.7%), sodium (23.1%), potassium (25.9%), creatinine (28.2%), and chloride (32.6%). Only jaundice was documented as a significant clinical abnormality with abnormal values of bilirubin.

**Figure 2:**
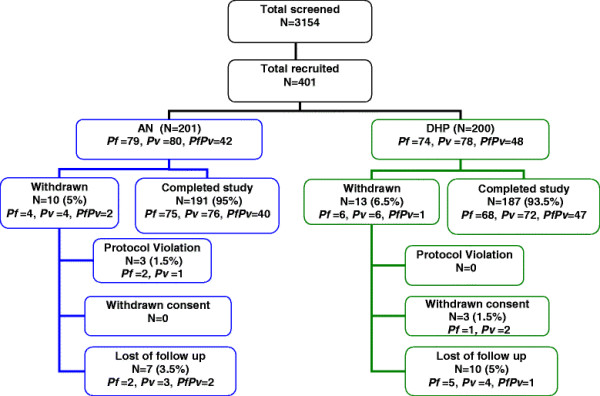
Clinical trial profile of artemisinin-naphthoquine (AN) versus dihydroartemisinin-piperaquine (DHP) groups.

There were 153 *Plasmodium falciparum* (*Pf*), 158 *Plasmodium vivax* (*Pv*) and 90 *P.falciparum/P.vivax* malaria (Figure 2). Gametocyte carriages were detected 34.4% (52 of 151) in *P. falciparum*, 94.3% (148 of 157) in *P. vivax* and 82.2% (74 of 90) in mixed *P.falciparum/P. vivax,* respectively. The range of gametocyte densities was 1–2,697/ul (Table 2).

### Analysed population

The ITT (401), modified ITT (384) and PP (378) population in each treatment group were microscopically cross-checked. There were three of 401 study subjects not eligible after cross-checking. All protocol violation cases were in AN group (two *P. falciparum* cases having asexual parasitaemia <1,000/ul, and one *P. vivax* case had taken an anti-malarial drug/chloroquine prior to the study). While all three withdrew consent, the cases were in DHP group (one parasitaemic *P. falciparum* case on day 0, one withdrawn *P. vivax* case by family of subject on day 0, and the one *P. vivax* case felt discomfort and dizzy by day 4). During the follow up, 17 cases were documented lost to follow up, seven (two *P. falciparum* cases on days 14 and 35; three *P. vivax* cases on days 3, 21, and 22; and two mixed *P. falciparum/P. vivax* cases on days 7 and 42) in AN group, and 10 [five *P. falciparum* cases on days 3, 7, 15, 20, and 21; four *P. vivax* cases on days 3 (two), 21, and 22; and one mixed *P. falciparum/P. vivax* case on day 3)] in DHP group ( 2 and Table 3).

**Table 3:** PCR uncorrected efficacy of AN vs DHP in all malaria, Indonesia

**Outcome**	**Artemisinin- naphthoquine (AN)**	**Dihydroartemisinin-piperaquine (DHP)**	**P**	**Overall**
**ACPR/Treatment Success (%, 95% CL)**				
**ITT**	179 [89.1 (84.7–93.4)]	180 [90.0 (85.8–94.2)]	0.76	359 [89.5 (86.1–92.3)]
**Modified ITT**	179 [92.3 (88.5–96.0)]	180 [94.7 (91.6–97.9)]	0.33	359 [93.5 (90.5–95.7)]
**Per Protocol**	179 [93.7 (90.3–97.2)]	180 [96.3 (93.5–99.0)]	0.26	359 [95.0 (92.3–96.9)]
**Late Clinical Failure-LCF (%, 95% CL)**				
**ITT**	5 [2.5 (0.3–4.6)]	1 [0.5 (0.5–1.5)]	0.10	6 [1.5 (0.6–3.2)]
**Modified ITT**	5 [2.6 (0.3–4.8)]	1 [0.5 (0.5–1.6)]	0.10	6 [1.6 (0.6–3.4)]
**Per Protocol**	5 [2.6 (0.4–4.9)]	1 [0.5 (0.5–1.6)]	0.10	6 [1.6 (0.6–3.4)]
**Late Parasitological Failure-LPF (%, 95% CL)**				
**ITT**	7 [3.5 (0.9–6.0)]	6 [3.0 (0.6–5.4)]	0.78	13 [3.2 (1.7–5.5)]
**Modified ITT**	7 [3.6 (1.0–6.2)]	6 [3.2 (0.7–5.6)]	0.81	13 [3.4 (1.8–5.7)]
**Per Protocol**	7 [3.7 (1.0–6.3)]	6 [3.2 (0.7–5.7)]	0.81	13 [3.4 (1.8–5.8)]
**“Other Failures” (%, 95% CL)**				
**ITT**	10 [5.0 (2.0–8.0)]	13 [6.5 (3.1–9.9)]	0.51	23 [5.7 (3.7–8.5)]
**Modified ITT**	3 [1.5 (0.2–3.3)]	3 [1.6 (0.2–3.4)]	0.98	6 [1.6 (0.6–3.4)]
**Per Protocol**	0	0		0

All protocol violations, withdrawn consents, and lost to follow up were classified as “other failures” in ITT analysis.

All protocol violations and withdrawn consents were classified as “other failures” in modified ITT analysis.

### Therapeutic efficacy

**Figure 3:**
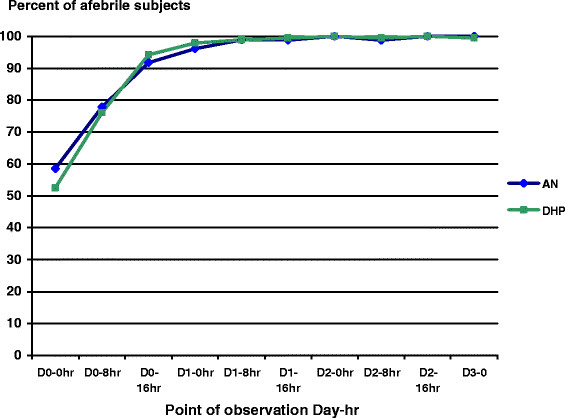
Proportions of afebrile malaria subject treated with artemisinin-naphthoquine (AN) versus dihydroartemisinin-piperaquine (DHP) groups were similar by point of observation (p > 0.05).

Both study drugs had rapid fever clearance. Over 90% of study subjects became afebrile by the first 16 hours after the first dose of treatment, and all cleared in 56 hours (Figure 3). The mean of fever clearance times (FCTs) were 13.0 ± 10.3 hours in AN and 11.3 ± 7.3 hours in DHP groups, and did not significantly differ.

Almost all hospitalized study subjects (>80%) were asymptomatic when discharged. During study follow-up, only mild reported symptoms (headache, dizzy, cough, abdominal pain, myalgia, sleeping disturbance, and fatigue) resolved with or without a simple symptomatic treatment.

**Figure 4:**
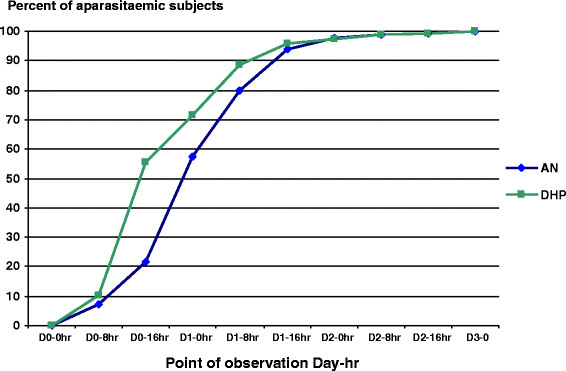
Proportions of aparasitaemic (asexual) malaria subject treated with artemisinin-naphthoquine (AN) versus dihydroartemisinin-piperaquine (DHP) groups were significantly difference by Day 0–16 hr (21.8% vs 55.6%, p < 0.0001), Day 1–0 hr (57.5% vs 71.3%, p = 0.01), and Day 1–8 hr (79.8% vs 88.5%, p = 0.03).

Most subjects (>90%) had cleared asexual parasitaemia by day 1–16 hours (Figure 4). AN had longer mean of parasite clearance time (PCT: 28.0 ± 11.7 hours) compared with DHP groups (25.5 ± 12.2 hours) (p = 0.04). Overall, mean of PCT of these ACTs was 26.7 (8–72) hours.

There was 68.8% gametocyte carriages prior ACT treatment. However, the proportion of gametocyte carriages reduced by day of follow-up. It became 28.7% and 25.8% in the first 24 hours post-treatment, and 18.6% and 17% by day 3 in AN and DHP groups, respectively. Gametocyte carriage was still detected in 1.1% subjects on completion of the study on day 42.

Of 401 randomized study subjects, there were 19 TFs, 12 in AN and seven in DHP groups. No ETF was reported. There were six documented as LCFs and 13 as LPFs (Table 3). All the TF cases were from Jayapura, four *P. falciparum*, three *P. vivax* and 12 *P. falciparum/P. vivax* malaria. Only three of 19 TFs occurred by day ≤28, two in the AN and one in the DHP group. PCR speciation of the 19 paired samples of TF cases showed three LPFs diagnosed as *P. vivax* (one in AN group by day 32 and two in DHP by day 35) detected as *P. falciparum*, and classified as protocol violation cases. There were another two LPFs by days 35 and 42 diagnosed as *P. falciparum*, one LPF by day 35 as mixed *P. falciparum/P. vivax*, and another one LCF by day 42 as mixed *P. falciparum/P. vivax* detected as *P. falciparum* new infections in AN group, Of the four TFs detected as new infections, two TFs had been treated with quinine plus doxycycline and classified as protocol violations at day 35. The PCR-corrected treatment outcomes are shown in Table 4.

**Table 4:** PCR corrected efficacy of AN vs Duocotecxin in all malaria, Indonesia

**Outcome**	**Artemisinin- naphthoquine (AN)**	**Dihydroartemisinin-piperaquine (DHP)**	**P**	**Overall**
**ACPR/Treatment Success (%, 95% CL)**				
**ITT**	181 [90.0 (85.9–94.2)]	180 [90.0 (85.8–94.2)]	0.99	361 [90.0 (86.7–92.8)]
**Modified ITT**	181 [93.3 (89.8–96.8)]	180 [94.7 (91.6–97.9)]	0.55	361 [94.0 (91.1–96.2)]
**Per Protocol**	181 [96.3 (93.6–99.0)]	180 [97.3 (95.0–99.6)]	0.58	361 [96.8 (94.4–98.3)]
**Late Clinical Failure-LCF (%, 95% CL)**				
**ITT**	4 [2.0 (0.1–3.9)]	1 [0.5 (0.5–1.5)]	0.18	5 [1.2 (0.4–2.9)]
**Modified ITT**	4 [2.1 (0.1–4.1)]	1 [0.5 (0.5–1.6)]	0.18	5 [1.3 (0.4–3.0)]
**3. Per Protocol**	4 [2.1 (0.1–4.2)]	1 [0.5 (0.5–1.6)]	0.18	5 [1.3 (0.4–3.1)]
**Late Parasitological Failure-LPF (%, 95% CL)**				
**ITT**	3 [1.5 (0.2–3.2)]	4 [2.0 (0.1–3.9)]	0.70	7 [1.7 (0.7–3.6)]
**Modified ITT**	3 [1.6 (0.2–3.3)]	4 [2.1 (0.1–4.1)]	0.68	7 [1.8 (0.7–3.7)]
**Per Protocol**	3 [1.6 (0.2–3.4)]	4 [2.2 (0.1–4.3)]	0.69	7 [1.9 (0.8–3.8)]
**“Other Failures” (%, 95% CL)**				
**ITT**	13 [6.5 (3.1–9.9)]	15 [7.5 (3.8–11.1)]	0.68	28 [7.0 (4.7–9.9)]
**Modified ITT**	6 [3.1 (0.7–5.5)]	5[2.6 (0.4–4.9)]		11 [2.9 (1.4–5.1)]
**Per Protocol**	0	0	0.79	0

All protocol violations, withdrawn consents, and lost to follow up were classified as “other failures” in ITT analysis.

All protocol violations and withdrawn consents were classified as “other failures” in modified ITT analysis. PCR corrected was carried out only for *P.falciparum* recurrences.

**Figure 5:**
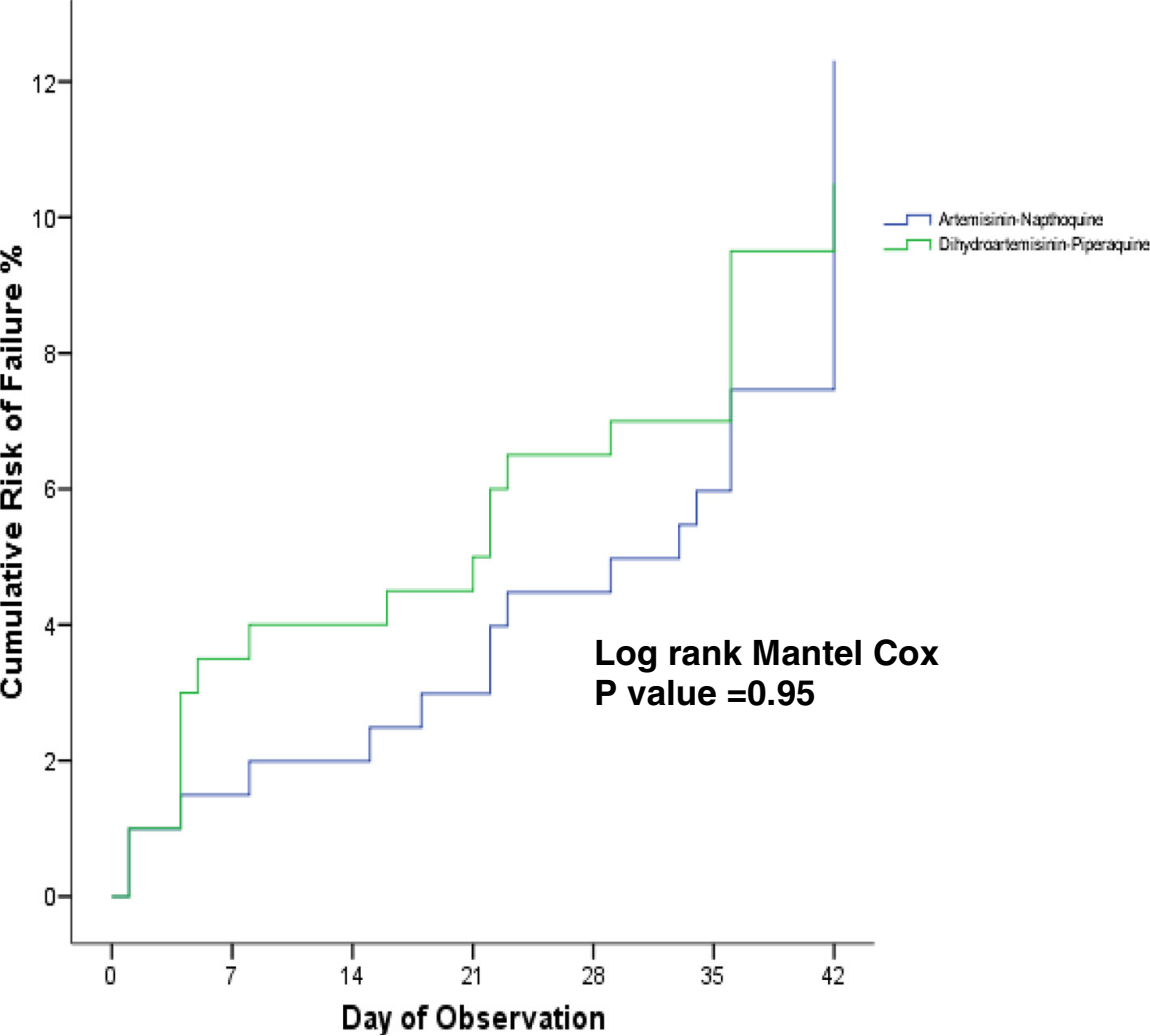
**Cumulative risk of failure of artemisinin-naphthoquine (AN) versus dihydroartemisinin-piperaquine (DHP) in ITT population infected with any malaria.** The TF by day 28 and 42 were 4.5% (9 of 201) and 10% (20 of 201) in AN group, and 7% (14 of 200) and 10% (20 of 200) in DHP group. The hazard ratio of failure between AN and DHP groups was 0.98 (95% CI: 0.53–1.82) with p = 0.95.

**Figure 6:**
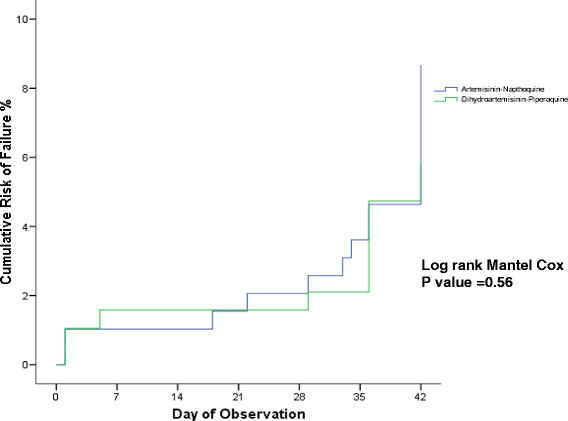
**Cumulative risk of failure of artemisinin-naphthoquine (AN) versus dihydroartemisinin-piperaquine (DHP) in modified ITT population infected with any malaria.** The TF by day 28 and 42 were 2% (4 of 196) and 6.7% (13 of 194) in AN group, and 2.1% (4 of 190) and 5.3% (10 of 190) in DHP group. The hazard ratio of failure between AN and DHP groups was 1.28 (95% CI: 0.56 – 2.92) with p = 0.56.

**Figure 7:**
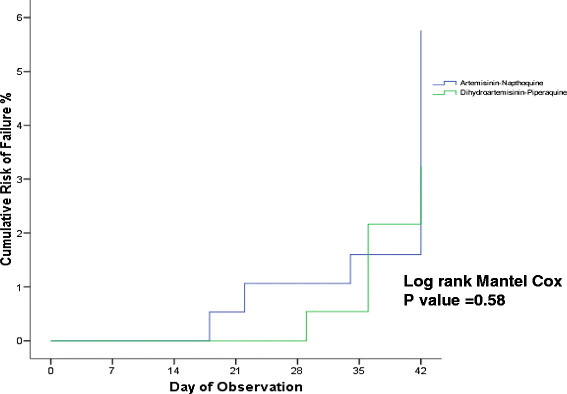
**Cumulative risk of failure of artemisinin-naphthoquine (AN) versus dihydroartemisinin-piperaquine (DHP) in PP population infected with any malaria.** The TF by day 28 and 42 were 1% (2 of 194) and 3.7% (7 of 188) in AN group, and 0.5% (1 of 187) and 2.7% (5 of 185) in DHP group. The Hazard ratio of failure between AN and DHP groups was 1.38 (95% CI: 0.44 – 4.35) with p = 0.58.

The overall uncorrected and corrected PCR therapeutic efficacies of both ACT for any malaria were between 89% to 95% in ITT and modified ITT population, and 94% to 97% in PP population. DHP had slightly higher uncorrected and corrected PCR at day 42 ACPR (96.3% and 97.3%) compared to AN (93.7% and 96.3%) for treatment of any malaria (Tables 3 and 4). All TFs’ PCR-corrected differences were detected in >28 day. Therefore, the day 28 uncorrected and corrected ACPR were similar, 95.5% (192 of 201) and 93.0% (186 of 200) in AN and DHP ITT populations; 98.0% (192 of 196) and 97.9% (186 of 190) in AN and DHP modified ITT population; 99.0% (192 of 194) and 99.5% (186 of 187) in AN and DHP PP population. Figures 5, 6 and 7 show the survival curves of PCR-corrected cumulative risk of failures of AN and DHP for treatment of any malaria in ITT, modified ITT and PP population. The hazard ratio of risk failures were no different between treatment groups in the three populations.

### Safety

There were no serious adverse events reported in malaria subjects treated with AN and DHP during the study. Only few (<10%) and mild symptoms as adverse events were documented. The common reported adverse events were headache, dizzy, and cough. There were also no clinically significant effects on myocardial electrophysiology identified through a series of ECG examinations.

In both study groups, haemoglobin, haematocrit, red blood cell (RBC) and platelet counts were gradually increased to normal limit by day 28. Means of haematocrit and RBC were slightly decreased on day 3, and 7, and became normal by day 28. In contrast, means of platelet on days 3 and 7 were increased significantly and then slightly decreased to normal value by day 28. The means of haematocrit, Hb, RBC, WBC and platelet at point of investigation were not statistical significant different between treatment groups. Only few leucocytosis (5.5%) were found in this trial. Interestingly, there were 21.4% eosinophilia (eosinophil >3%) on day 0, and increased to 38.5% by day 28. All the abnormal haematological values had no significant clinical abnormality.

There were mild liver impairment with or without mild renal impairment on day 0 however bilirubin, albumin, ALT, AST, creatinine, urea and electrolytes were gradually improved to normal limit by day 28 in malaria subjects treated with AN and DHP. The means of these blood chemistry parameters were not different between treatment groups.

## Discussion

ACT is recommended to be given for 3 days when given with slowly eliminated partner drug. In a three-day regimen, the artemisinin component is present in body during only two asexual parasite life-cycles, except for *Plasmodium malariae*. In each asexual cycle, artemisinin and its derivatives reduce parasite numbers by a factor of approximately 10,000 [2,12]. This anti-malarial drug is a potent and rapidly acting blood schizontocide, and gametocytocide [5,7]. It is still rational to study one-day ACT with a new good partner drug to improve the compliance and have a simple and practical regimen. Moreover, the risk of the development of de novo resistance is increased by the greater time dividing asexual parasites are exposed to drugs. This makes the long-term risk of resistance developing a concern for single dose ACT. Therefore, this study will be useful to confirm the findings of other previous studies with different types of one-day ACT (artesunate-amodiaquine, artesunate-sulphadoxine/pyrimethamine, or artesunate-mefloquine) [18,19] and in different geographical settings.

Naphthoquine is a tetra-aminoquinoline, synthetic blood schizontocide anti-malarial drug. Though this anti-malarial drug has a similar structure to chloroquine, cross-resistance with chloroquine has not yet been reported. Of the limited clinical studies in China, naphthoquine in combination with artemisinin given in a single dose, was effective and safe with cure rates of 97.5% for treatment of *P. falciparum* malaria by day 28, and 90.0% for the treatment of *P. vivax* malaria by day 56 [11]. This preliminary findings is valuable data and a good start for clarification whether the three-day regimen ACT is mandatory.

Of the existing forms of ACT which had been studied in Indonesia, a three-day dihydroartemisinin-piperaquine (DHP) is the best alternative for the Indonesian ACT programme. DHP is safe and effective for both *P. falciparum* and *P. vivax* malaria. The cure rates of DHP for the treatment of *P. falciparum* and *P. vivax* were reported 95.2% and 92.7%. While the cure rates of AA, the first ACT programme were only 84% and 53.5% [6]. In addition, DHP had post treatment prophylactic effect in high transmission area [7]. DHP is a good comparator for a trial of a new ACT.

Currently, multiple first-line therapies (MFT) policies against malaria have been introduced. It was developed in the context of an evolutionary-epidemiological modelling framework for malaria resistance. The benefits of using MFT against malaria yields a better clinical outcome, delay the emergence and slow the fixation of resistant strains, and allow a larger fraction of the population to be treated without trading off future treatment of cases that may be untreatable because of high resistance levels [20]. Despite DHP, other forms of ACT should be identified to be chosen for MFT policies, including AN.

In this trial, one-day single dose of fixed dose regimen of AN and three-day regimen of DHP did not result ETF for treatment of any malaria. All 19 TFs were as LTFs and reported from Jayapura only. Of the 19 TFs, 63.2% were related with clinical symptoms and classified as LCFs. Though 84.2% TFs were occurred by day >28, only 21.1% TFs (4 of 19) confirmed as new infections which were detected by days 35 and 42. This findings support Papua as a highly multidrug resistance area. A significant bigger number of samples from Jayapura (75% of a total sample) probably gave a chance detected more TF cases. Moreover, a relatively small number of new infections in a moderate-high transmission Papua by days 35 and 42 could be because of long half-life of both study drugs AN and DHP which known have post treatment prophylactic effect [6,7]. ACT with a long half-life partner drug is a good choice for malaria in high transmission area, however there will be also an increased risk of selecting drug-resistant isolates [21]. Therefore, monitoring drug efficacy should be routinely maintained to detect the spread of drug resistance.

In this trial, both drugs showed very effective for treatment of any malaria. AN and DHP had day-42 PCR corrected ACPR of ≥90% in ITT and modified population, and >95% in PP population. Moreover, the day-28 and day-42 ACPRs of AN and DHP were not statistically different (p > 0.05). These findings are consistent with previous efficacy studies of AN for treatment of uncomplicated *P.falciparum* malaria in China [11], Myanmar [22] and Papua New Guinea [23], and DHP for treatment of uncomplicated *P.falciparum* and *P.vivax* malaria in Indonesia [6,7] and for treatment of uncomplicated *P.falciparum* malaria in other Asian countries [24-28].

Similarly to other forms of ACT, AN and DHP cleared fever and asexual parasites rapidly with means FCT 13.0 and 11.3 hours, and PCT 28.0 and 25.5 hours. These findings were similar to the previous studies [6,7,11,22-28]. Both forms of ACT also resulted 66.5% haemoglobin recovery by day 28. The anaemic study subjects with Hb < 11 g% were significantly decreased from 23.6% and 21.6% prior treatment and became 5.9% and 9.9% by day 28 in AN and DHP groups. Though many factors influence the recovery of haemoglobin, a long half-life of partner drugs naphthoquine and piperaquine have an important role to prevent re-infections or relapses, which might cause anaemia.

Both AN and DHP were well-tolerated, with no significant ECG changes identified. Most of the adverse events were mild and related with symptoms attributable to malaria. All symptoms were recovered with or without simple treatment. There were no significant differences in tolerability between the two study drugs.

In this clinical trial, eosinophilia was found in one third of study subjects treated with AN or DHP. Several studies also reported that pseudoeosinophilia was associated with malaria infection detected by Sysmex XE-2100 haematology analyzer due to the presence of haemozoin-containing neutrophils [29,30]. Eosinophilia may also represent a normal late response to malaria infection [31,32]. However, all eosinophilia subjects had no significant clinically abnormality. There were also slightly elevations of aminotransferase (ALT and AST) in study subjects treated with AN and DHP which gradually decreased to normal limit. These findings were reported similar to Chinese studies [33].

This study has shown the efficacy and safety a single dose of AN and a three-day dose of DHP for treatment of any malaria in adult subjects. Further analysis of the efficacy and safety specifically in *P. falciparum* and *P. vivax*, and others malaria should be performed and published to show the detail of study findings. In addition, there was a study reported a high cure rate of twice daily one day of AN (100%) versus AL (98.4%) in African children with *P. falciparum* malaria [34]. The dosage used in that study was based on the children weight groups. Dosage for children is crucial and will be safe determined by per kg body weight [35]. Cost of drug is also important factor for choosing programme malaria drug. A single dose ACT, such as AN actually should be cheaper compare with three-day regimen ACT. Moreover, there are study limitations to extend follow up day to D42 whereas D63 is recommended for AN with a long half lives, and the small sample size and consequent loss of power.

## Conclusions

Both fixed-dose forms of ACT are confirmed very effective, safe and tolerate for treatment of any malaria in adults, and meet with the recent WHO recommendation for replacing ineffective drugs. Their longer post-treatment prophylactic effect is useful in areas where transmission is intense. Artemisinin-naphthoquine and dihydroartemisinin-piperaquine are promising forms of ACT for MFT policy. Pharmacokinetic and therapeutic efficacy study in children, and cost-effectiveness study should be carried out for the safety and effectiveness of large-scale use.

## Competing interests

The authors declare that they have no competing interests.

## Authors’ contributions

ET: study conceptualization and design. ET, ARH, HS, BP, RE, EAY, TP, SL, ES, N, EY, LY, JL, BW, FA, TADD, AP performed the study. ET, ARH, HS and ES analysed the data. ET, ARH, ES, T prepared the manuscript. All authors have read and approved the final manuscript.

## Funding

The study was funded by the Kunming Pharmaceutical Corporation.
